# Engaging Men in Family Planning: Perspectives From Married Men in Lomé, Togo

**DOI:** 10.9745/GHSP-D-17-00471

**Published:** 2018-06-27

**Authors:** Tekou B. Koffi, Karen Weidert, Eralakaza Ouro Bitasse, Marthe Adjoko E. Mensah, Jacques Emina, Sheila Mensah, Annette Bongiovanni, Ndola Prata

**Affiliations:** a Cabinet de Recherche et d'Évaluation (CERA), Lomé, Togo.; b Bixby Center for Population, Health and Sustainability, School of Public Health, University of California at Berkeley, Berkeley, CA, USA.; c University of Kara, Kara, Togo.; d International Business and Technical Consultants, Inc. (IBTCI), Vienna, VA, USA.; e University of Kinshasa, Kinshasa, Democratic Republic of the Congo.; f United States Agency for International Development/West Africa, Regional Health Office, Accra, Ghana.

## Abstract

Family planning programs have made vast progress in many regions of sub-Saharan Africa in the last decade, but francophone West Africa is still lagging behind. More emphasis on male engagement might result in better outcomes, especially in countries with strong patriarchal societies. Few studies in francophone West Africa have examined attitudes of male involvement in family planning from the perspective of men themselves, yet this evidence is necessary for development of successful family planning projects that include men. This qualitative study, conducted in 2016, explored attitudes of 72 married men ages 18–54 through 6 focus groups in the capital of Togo, Lomé. Participants included professional workers as well as skilled and unskilled workers. Results indicate that men have specific views on family planning based on their knowledge and understanding of how and why women might use contraception. While some men did have reservations, both founded and not, there was an overwhelmingly positive response to discussing family planning and being engaged with related decisions and services. Four key findings from the analyses of focus group responses were: (1) socioeconomic motivations drive men's interest in family planning; (2) men strongly disapprove of unilateral decisions by women to use family planning; (3) misconceptions surrounding modern methods can hinder support for family planning; and (4) limited method choice for men, insufficient venues to receive services, and few messages that target men create barriers for male engagement in family planning. Future attempts to engage men in family planning programs should pay specific attention to men's concerns, misconceptions, and their roles in family decision making. Interventions should educate men on the socioeconomic and health benefits of family planning while explaining the possible side effects and dispelling myths. To help build trust and facilitate open communication, family planning programs that encourage counseling of husbands and wives in their homes by community health workers, trusted men, or couples who have successfully used or are currently using family planning to achieve their desired family size will be important.

## Abstract (precis)

Men in the study generally supported couples' use of contraception, especially citing socioeconomic reasons. Some had reservations stemming from perceptions that family planning could facilitate infidelity and promiscuity. They also thought family planning decisions should be made jointly. All men expressed interest in learning more about family planning, preferring dissemination from community health workers, trusted men, and current family planning users.

Résumé en français à la fin de l'article.

## INTRODUCTION

During the last decade, family planning activities have resulted in increased voluntary uptake of family planning in many regions of sub-Saharan Africa, yet high fertility and low contraceptive prevalence persist in francophone West Africa.^1^^,^^2^ Recognizing this lag, representatives from francophone West African countries gathered in Ouagadougou, Burkina Faso, in February 2011 to discuss initiatives that address population growth, climate, and family planning. Participating countries, which included Benin, Burkina Faso, Côte d'Ivoire, Guinea, Mali, Mauritania, Niger, Senegal, and Togo, formed the Ouagadougou Partnership, with a call to action through the Ouagadougou Declaration to strengthen family planning services, mobilize political commitment and resources, and coordinate actions.^3^ The Ouagadougou Partnership paved the way forward in francophone Africa at the same time that Family Planning 2020 (FP2020) was gaining momentum. Member countries of the Ouagadougou Partnership agreed to reach at least 1 million additional women in the region with voluntary family planning services by 2015.^4^ All member countries also worked with FP2020 to develop Costed Implementation Plans (CIP), which are multiyear roadmaps to help governments achieve their family planning goals by prioritizing interventions, engaging stakeholders around one strategy, forecasting costs, and mobilizing resources to meet any gaps.^5^

Given the success of the Ouagadougou Partnership in reaching its target of an additional 1 million contraceptive users by 2015, ministers of health from the member countries reconvened to develop an accelerated plan for 2016–2020, which included a new target of reaching an additional 2.2 million women with voluntary contraceptive services. The member countries also agreed to review and revise their action plans, taking into account scientific evidence and promising innovations. There is a general acknowledgment that these revisions posed both opportunities and challenges, given limited availability of research and evidence on family planning programs in francophone West Africa.^6^ That said, given the primary role of men in decision making in francophone West Africa, engagement of men in family planning should be a critical component of the revised action plans.

Male engagement should be a critical component of francophone West African countries' family planning programs.

### Togolese Context

The latest Togo Demographic and Health Survey (DHS), conducted in 2013-14, reported a total fertility rate (TFR) of 4.8.^7^ FP2020, in its 2016-17 annual report, measured low modern contraceptive prevalence among all women at 23.2% and high unmet need among married women at 34.6%.^8^ Meanwhile, in the 2013-14 DHS, 57% of women reported their husband was the main decision maker for their own health care, whereas 82% of men reported they solely made decisions surrounding their own health care. At the same time, 52% and 71% of women and men, respectively, reported that men make most decisions surrounding major household purchases. Ninety-five percent of men reported having final say in making both types of decisions.^7^

As part of repositioning family planning between 2012 and 2015, Togo improved access to family planning services by implementing a number of activities, including free family planning services through mobile strategies and special days, community-based distribution of contraceptives including injectables with the support of NGOs and associations, and authorization for setting up mobile clinics through NGOs and associations.^9^ The Government of Togo also committed to the following targets after the Family Planning Summit in July 2017 in London^10^:

1. Develop a new plan to accelerate family planning access in Togo with the aim of increasing modern contraceptive prevalence among women in union from 23% in 2017 to 35.5% in 2022
2. Quadruple the annual state subsidy for the purchase of contraceptive products between 2016 and 2022
3. Ensure the implementation of comprehensive sex education for adolescents and young people in all schools by 2022
4. Improve the supply chain of family planning products by reducing stock-outs of contraceptives at service delivery points by 50% between 2017 and 2022

While these commitments and ongoing efforts are important and necessary, the role of men is not fully addressed. The engagement of married men in family planning will be critical to achieving the ambitious national goals, particularly in light of the decision-making power yielded by men in Togo combined with their historically low engagement in family planning.^9^

### Male Engagement in Family Planning

Because women are the ones who face the risks associated with pregnancy and childbirth, they are often the focus of family planning programs. Furthermore, most contraceptive methods are female-controlled, giving women better control over their fertility. Programs presume that women have greater motivation than men to use family planning services, and they usually interact more with health care services in general than men. Yet this targeted programming often overlooks the gender-related power dynamics that position men at the head of the household with decision-making power, including whether and when sex occurs and if contraception is used.^11^

There are several proven, promising, and emerging strategies to engage men as contraceptive users. Male motivators, social marketing, and mHealth have been identified as proven strategies, whereas comprehensive sexual education, community dialogue, and clinic provision of information are designated as promising strategies.^12^ The Ministry of Health in Togo has reported achievements with their Committees of Men project,^9^ which was inspired by the Husband Schools project in Niger^13^ where men are engaged to promote reproductive health and behavioral change in their communities. In fact, the ministry planned to scale up this project between 2013 and 2017.^9^ The success of this project was likely highlighted by participants in another recent study assessing how to best engage men in Togo who mentioned that exchanges with “Papa Champions,” men who are already strongly engaged in the health of their families and recognized in the community, was a strategy for improving male involvement in family planning.^14^

In the realm of male motivators, positive deviance has also emerged as a preferable approach to increase awareness of family planning among men, including in another study from Togo.^14^ This is a bottom-up approach that focuses on the individuals in every community who behave differently—their uncommon (positive) practices, such as using contraception consistently or communicating with his spouse about family planning—enable success in areas where their neighbors fail. The approach seeks out these “positive deviants” in the community and uses their existing solutions to bring about sustainable behavioral and social change.^15^ While the positive deviance approach has had limited application in family planning programs to date, it is feasible and logical in the context of using community-based approaches to increase awareness and use of modern methods, particularly among men with unfounded concerns.

Engaging religious leaders, which is considered an emerging strategy, has had success in francophone West Africa. In 2013, the Senegal Ministry of Health and Social Action launched the first-ever national family planning communication campaign addressing men as a primary target audience. Muslim religious leaders were included among the messengers and influencers represented in the campaign. After the campaign, a survey was conducted with 1,800 men responding. The findings showed that more than 68% initiated a discussion with their partner about family planning and more than 12% reported that their partner now practices family planning as a result of the campaign.^16^ This case is not unique, as across the region, religious leaders are emerging as champions and speaking in favor of family planning to ensure the healthy timing and spacing of pregnancies. In fact, Togo, in its 2013–2017 plan to reposition family planning, committed to training religious leaders on the advantages of family planning,^9^ enabling them to deliver sermons on family planning and also establishing a cadre of family planning champions who could train their peers of other religious leaders.

Though the examples above provide evidence of promising interventions, there is a continued need for evidence on how to engage men in family planning in francophone West Africa. Failure to involve men in family planning can have important implications, even when women are educated and motivated to use contraception.^17^ It is also well documented that men's general knowledge and attitudes related to family size, spacing between children, and contraceptive methods affect women's family planning preferences and opinions.^18^^–^^20^ DHS data from 7 African countries, including Togo, revealed that the percentage of women who used modern contraceptives was higher among those who had discussed family planning with their spouses than among those who did not.^21^ A 1996 study in Togo found the likelihood of spousal communication about family planning and modern contraceptive use was significantly higher among women who exercised complete control over the selection of their partner than among those with arranged marriages. Additionally, women who worked for cash were significantly more likely than those who did not to communicate with their spouses about family planning, particularly if they participated in rotating credit or savings schemes. Such participation also significantly increased the likelihood of ever using traditional or modern methods of contraception.^22^

There is continued need for evidence on how to engage men in family planning in francophone West Africa.

Many scholars have argued that the inability to engage men in family planning is impeding progress to increase uptake of contraception.^19^^,^^23^^–^^26^ The role of men in family planning has been seen as crucial for optimizing family planning services. A review of existing studies of male involvement in family planning in sub-Saharan Africa found that among the reasons for poor male involvement in family planning were cultural barriers, such as embarrassment with visiting family planning service delivery points.^20^ The review also found that poor male involvement in family planning was associated with poor communication between men and their female partner, although a majority of men disagree that they should be the sole family planning decision makers. Finally, it was found that definitions of male involvement in family planning have varied, making it difficult to compare the results and efficacy of the few interventions that have been implemented.^20^ These challenges point to the need for further evidence generation on male engagement in family planning. Few studies have been carried out to understand the barriers to male involvement in family planning from the male perspective in francophone West Africa. This is in spite of policy trends in the region, which encourage men to take more responsibility for family planning and parenting.

### Purpose of the Study

The purpose of this study was to gain insights on how to best engage men in future family planning programs in Lomé, Togo. Through male perspectives, we assessed their experiences, perceptions, and preferences, specifically with regard to spousal use of contraception, family planning discussions, and how men would like to receive family planning information and services. Results from this study might have implications for a broader audience in urban and peri-urban Togo, as well as for similar settings in francophone West Africa.

## DATA AND METHODS

### Study Design

A qualitative study was conducted in 2016 using focus group discussions with men ages 18–54 in urban and peri-urban areas of Lomé, Togo. Data used in this study were collected as part of operations research conducted in Lomé to assess the effectiveness of an ongoing family planning service delivery model of the *Agir pour la Planification Familiale* (AgirPF) program of the U.S. Agency for International Development (USAID)/West Africa and EngenderHealth.^27^ The goal of AgirPF is to enable women of reproductive age to make, and voluntarily act on, informed decisions about family planning, saving women's lives in selected urban and peri-urban areas of 5 francophone West African countries: Burkina Faso, Côte d'Ivoire, Mauritania, Niger, and Togo. AgirPF incorporates many high-impact practices in its technical approach,^28^ including a range of program activities from addressing quality of clinic services and provider training to the availability of mobile services and community-based distribution.^27^ More specifically, to bring family planning services to underserved communities, AgirPF supports mobile outreach services, brings health fairs to industries and community sites, offers “city-based services,” an adaptation of EngenderHealth-managed community-based distribution in Togo, and supports community leaders with family planning advocacy activities.^27^

### Participant Selection

Men who were married or cohabiting and residing in AgirPF program catchment areas were eligible to participate. Advertising and recruitment were carried out by local development organizations and NGOs working with the AgirPF program. Participants were recruited from 3 health districts (District II, District III, and District V) and Maritime Health District (Golfe). Site selection for the focus group discussions was based on the site selection for the larger study to assess AgirPF program. We conducted 2 focus group discussions in District III and Golfe District and 1 focus group discussion each in Districts II and V. No personal information in the form of names or other identifying data was obtained. All participants gave informed consent and the study was approved by the Republic of Togo Ministry of Health and Social Protection, Bioethics Committee for Research in Health (AVIS N0 017/2016/CBRS du 30 Juin 2016) and the University of California Berkeley Committee of Protection of Human Subjects (CPHS #2016-04-8614).

### Data Collection

A total of 6 focus groups were conducted in August 2016. Each focus group consisted of 12 men and averaged about 1 hour and 45 minutes. Open-ended, semi-structured question guides were used to explore perceptions regarding preferences and barriers to male involvement in family planning. Focus group discussions were conducted in French or a combination of French and a local language, and were audio-recorded with permission from the participants. They were led by a local team (men and women) of experienced qualitative investigators. All study participants were encouraged and given opportunities to openly discuss their opinions in confidence.

### Data Analysis

All discussions and interviews were recorded and transcribed verbatim in French and local languages and translated to English using thematic content analyses. A thematic analysis was conducted using deductive coding process. First, 2 coders reviewed the transcripts several times to identify major themes and develop a broad coding scheme based on the research questions. The codes were refined and emerging themes discussed and confirmed with the local research team. The coding team met regularly to discuss any discrepancies in code definitions and in the application of codes, ensuring the reliability of coded data and consistent coding of transcripts.

Focus group participants were asked their occupation and age during a sign-in procedure when they provided consent to participate. The participants were also assigned a number at check-in for which they would be referred to in transcripts, rather than their names. The transcripts included a key that indicated the age and occupation associated with each participant number. This allowed the coding team to assign an age and occupation when including quotations. However, there were not enough cases to stratify the analysis based on age and occupation.

## RESULTS

Among the 72 married men included in the focus groups, 20% were 30 years of age or younger, 53% were between ages 31 and 45, and the remaining 26% were over 45 years of age. They encompassed qualified professional workers (including a university professor, computer scientist, and journalist) and skilled and unskilled workers (including a driver, mason, and hairdresser). Major themes that emerged from the data were related to explanations for positive or negative views of family planning; views on spousal communication about family planning; interest in learning about family planning; views of current family planning services; and the role of male-controlled contraceptive methods including perceived barriers to male involvement in family planning.

We conducted focus group discussions with 72 married men to gain insights on how to best engage men in future family planning programs in Lomé, Togo.

### General Views on Family Planning

There were 3 groups of men in the sample based on their perceptions about family planning: (1) those who believed in family planning; (2) those who believed in family planning but had reservations about it; and (3) those who did not believe in family planning (Figure). Focus groups were mixed vis-à-vis these 3 categorizations and we specifically did not assign values related to number of men that fell into each category due to the purposive nature of the sample and our desire for the categories to be illustrative of the range of perceptions.

**FIGURE:**
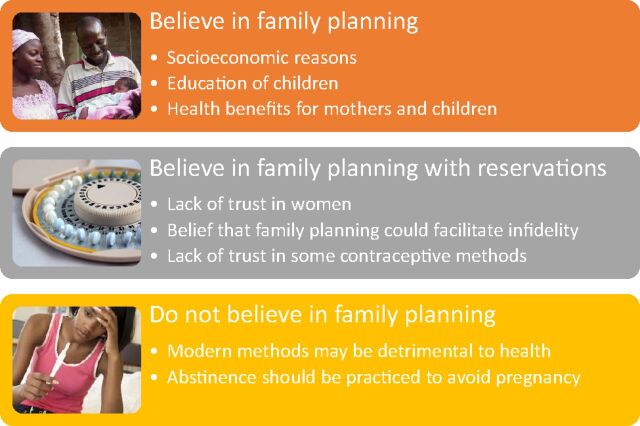
Three Categories of Male Focus Group Participants Based on General Views of Family Planning, Lomé, Togo, 2016

The men who believed in family planning often stated socioeconomic reasons associated with raising children, including the cost of their education, and the maternal and child health benefits of birth spacing as their main reasons for supporting family planning.

Men who believed in family planning often stated socioeconomic reasons for supporting it.

*Birth spacing is a good thing; this means when we bring a child into this world, the period of spacing between the second and third birth allows the mother and the child to recover to its [her] initial health. In addition, the mother is a bit free for other chores. –* 48-year-old teacher

Reservations about family planning seemed to stem from men's lack of trust of women and their views that family planning could potentially facilitate infidelity, promiscuity, and/or lead to commercial sex work. As one respondent put it:

*… this means that there are certain women whose husbands have left the country to immigrate to the West. Because they [the women] have sexual desire, they go to a provider to benefit from a contraceptive method with the intention not to get pregnant, this way they practice prostitution. –* 28-year-old merchant

Other reservations were linked to health concerns associated with specific contraceptive methods. Fear of side effects, threats to future fertility, risks to maternal and child health after contraceptive use, and misconceptions regarding the dispensing of contraceptive methods were all cited as reasons why one may avoid using family planning methods. Many men in this group believed that laboratory tests, including various blood tests, must be carried out before a contraceptive method is prescribed. There was a general perception that without specific tests, women will get the wrong method, which might end up affecting their health and their future children's health. This concern was best explained by one participant:

*… we notice that after the use of a method, when the method expires, women develop many health problems. When the method is removed, the same we find that the children born after the use of a method are not really in good health.* – 35-year-old entrepreneur

Non-believers in family planning often suggested that periodic abstinence could be practiced to avoid unintended pregnancies. They believed that was what their ancestors practiced in the absence of modern family planning. They associated sex only with reproduction, consequently believing that if a couple is not trying to conceive they should not have sex at all. Therefore, modern family planning methods were unnecessary, these men expressed.

*… our parents did not use any product. The understanding among couples is to practice birth spacing. If, as a man, there is no consent between the woman, and the man lays with the woman because of desire, that will only bring unintended pregnancies. Our grandparents themselves used this consent as the reason why they did not have unintended children.* – 37-year-old merchant

### Views Related to Use of Contraception

Independent of the categorization of participants mentioned above, the majority of men participating in the focus group discussions responded positively when asked about their views on couples who used contraceptive methods to space or limit pregnancies, noting it was the “good” and “responsible” thing to do. Though men were asked specifically about using contraceptive methods, they often responded with more general perspectives surrounding family planning. Common themes that arose during these discussions were that by practicing family planning, couples could make better use of limited resources and improve overall family wealth. Participants often cited that spacing and limiting births resulted in happier, healthier families and better relationships within the family. Other less prevalent benefits mentioned were maternal health and child well-being. More specifically, some men noted that birth spacing gives women appropriate time to recover between children. They also mentioned that birth spacing gives parents more opportunity to focus on their child and results in better prospects for education.

*A planned family is a happy family since we do not see them go into debt, running back and forth looking for a way out. They are free and always go about their activities. It is a model family.* – 40-year-old psychosocial counselor

*Let me say that couples who use contraceptive methods are healthy even if they do not have money. Their children grow well and they are content with the few resources they have.* – 30-year-old mobile phone repairman

However, not all responses were positive. Some men mentioned concerns with side effects of contraceptive methods and occasionally repeated a common misconception about long-term negative health impacts of contraception.

*[referring to perceived side effects of implant, earlier noting infertility and early menopause.]… expenditures for its treatment in the hospital and the treatment of other long-term side effects may exceed the costs related to the care of 10 children. Those couples, who today are victims due to contraceptive methods, will suffer more in the long term from other diseases.* – 42-year-old teacher

Most men had positive views of couples who used contraception to space or limit pregnancies.

### Views on Spousal Communication About Family Planning

While men largely spoke favorably about couples' use of contraception, the majority had negative, and often animated, responses when asked about a woman who uses contraception without the knowledge or authorization of her spouse. Many men noted that a woman does not have the right to make this decision alone and it would be grounds for divorce, explaining their suspicion of infidelity when a woman is using a family planning method secretly. In addition, some men had the perception that a law exists that prevents providers from distributing contraceptives to women without spousal consent.

Many men noted that a woman does not have the right to make the decision to use family planning on her own and that it would be grounds for divorce.

*There are many risks. Women who individually take the initiative to go for family planning methods without the knowledge of their partners are exposed to risks such as divorce, for example. Why not seek the advice of their husbands before going? The issue of procreation is decided by both partners. A woman has the right to do whatever she wants with her body; however, from the moment she gets married, the issue of having children or not, spacing births or not, should be consensually decided with the husband.* – 35-year-old PhD student

*Such things always lead to divorce in households in our community here. It is the man who is the head of the family. The law does not normally authorize the service provider to administer a method to a woman without consent of her husband.* – 48-year-old teacher

Despite a general sentiment supporting spousal consent for a woman's contraceptive use, some men did mention cases whereby such behavior would be justified by women. During the focus groups, men discussed understanding a woman's motivation for using contraception without the knowledge of her husband. A small number of participants felt it was appropriate if the woman had a psychological or medical reason or was responsible for all the household expenses.

*In reality, my wife had the implant without my knowledge and I only found out after two years. Indeed I was traveling, and after a sensitization session in the neighborhood, she opted for the implant. And when I discovered this, I was really angry with her before calming myself down. [But] after investigations [it was] revealed there was not hidden motive [in my wife's decision to use the implant].* – 30-year-old printer

*Yes, I don't blame the woman who used contraception without the knowledge of her husband. There is always a beginning [reason] for everything. We should try to understand whether the woman has enough children and doesn't want more.* – 37-year-old communicator

While less prominent, the theme of infidelity as a reason for covert contraceptive use also came up in discussions. A few men proposed that a woman covertly using contraception suggested promiscuity. Several men agreed that a women's covert use of contraception justified infidelity on the part of the man who is seeking more children.

*I have personally been a victim of this situation. As I insisted on making another child, I began to have another partner and my wife finally left the marital home.* – 28-year-old trader

Several men also stated that it was the provider's fault in cases where women were using contraception without the knowledge of their husbands.

*First of all, the error comes from the provider because this must be included in the information or sensitization [about contraception]; since there must be consensus between the man and the woman before the woman uses contraception. The provider should be sure of the husband's agreement before offering this service to the woman.* – 30-year-old barber

It was clear that the participants' general opinion was that family planning should be a decision made by a husband and wife. When asked how they felt about men who discuss family planning with their wives, most participants spoke positively of these men referring to them as “wise,” “thoughtful,” and “responsible.”

*He is a responsible man and a model for society because not all men will like to discuss such issues with their wives.* – 37-year-old communicator

They felt that having such discussions demonstrates strong and open relationships, whereas an inability to discuss family planning signals greater marital discord.

*The fact that the man discusses the issue with the wife is an indication of the good dialogue and communication which already exists between them.* – 29-year-old sociologist

*I think the couple is really solid. Sitting and making decisions together will make them strong and help them address many issues in the family.* – 34-year-old teacher

Moreover, the responses were overwhelmingly positive when participants were asked about discussing family planning with other people outside of the marriage. Most men suggested that such conversations occur when they are already discussing problems with friends and family, because these conversations provide an opportunity to give advice or elaborate on personal experience, which can include family planning. Some men mentioned bringing up birth spacing when having a discussion with someone who mentions their financial woes.

*If I am well informed about family planning and I practice it and it suits me, I can talk to my friend and suggest that he tries it too. In doing so, I train him and indirectly train society.* – 49-year-old printer

However, some participants remarked they would need training to feel comfortable discussing family planning, while others felt these discussions should be initiated by health care providers or peer educators who have been trained and have comprehensive, accurate information about family planning.

*Only those who have been trained can have relevant information about family planning since they are familiar with all the issues relating to it.* – 38-year-old evangelist

Men generally thought family planning decisions should be made jointly by a husband and wife.

### Interest in Learning About Family Planning

Regardless of their initial views on family planning, all men participating in the focus groups said they wanted to learn more about it. When probed on how, when, and where they would prefer to learn more about family planning, their suggestions spanned from identifying who should be providing information, venues for sharing information, and different channels through which information could be shared.

All the men wanted to learn more about family planning.

The majority of participants mentioned that the best way to share information would be door to door. Trained community health workers were considered a reliable source of initial information that would be disseminated by word of mouth in communities. Many venues were cited as important points where information could be disseminated including community gatherings, markets, clubs, popular community celebrations, the work place, and other media outlets. Men emphasized the importance of the credibility of those providing the information and accuracy of information. An organization considered credible for informing men on family planning issues was the Togolese Association for Family Wellbeing (ATBEF), an International Planned Parenthood Federation affiliate, as well as AgirPF. Although less frequently, participants also mentioned village development committees as avenues to spread information and football matches as a potential venue where information on family planning could be shared. Some men questioned the value and effectiveness of using health facilities as a means to inform men about family planning. Many reasons were cited including that many men do not go to the health facility; facilities are usually busy with providing care to sick patients; information is provided in sessions designed for women at facilities; and most providers are female, which may inhibit men's ability to comfortably ask questions and/or have an open discussion on contraceptive methods.

Focus group participants said they would like family planning messages to emphasize contraceptive side effects and the importance of smaller families for the health and well-being of the family. Overall, participants seemed to appreciate learning about family planning through sketches or cartoons, including those that resemble comic books. More importantly, they expressed a desire to talk with couples who had successfully used or were currently using family planning to achieve their desired family size—that is, positive deviant case studies—as well as those who did not use family planning resulting in a higher-than-desired family size.

*For me, I prefer to go with my wife to couples whose family planning practices have succeeded because I do not know if the provider himself has already used a family planning method, so I prefer to contact someone who has already practiced it.* – 38-year-old evangelist

Men expressed a desire to talk with couples who had successfully used or were currently using family planning.

### Views on Current Family Planning Services and the Role of Existing Male-Controlled Methods

Men discussed their views on current family planning services, including information campaigns, in part to justify their limited engagement in family planning thus far. Limited availability and understanding of male-controlled methods, as well as the way they perceived the family planning campaign in Lomé, were all critical themes to the conversation. Male condoms, vasectomy, and abstinence were identified by all focus group participants as male-controlled methods. Only one focus group also identified withdrawal as a male-controlled method. Male condoms were discussed as an important method but also “not for all”; some men remarked that women did not like condoms, while others did support their use. All participants were opposed to vasectomy, with the most notable reason for opposition being its irreversibility. Some believed that male performance would be negatively impacted.

*… I will say that this male method [vasectomy] is not good at all because we can no longer father a child. However, the ones [contraceptives] for women allow her to have a child when she wants. I cannot use this method of contraception, it will prevent me from having children. That is why I can never opt for this method.* – 30-year-old mobile phone repairman

All of the men were opposed to vasectomy, mostly because it was irreversible.

Abstinence was positively mentioned by all participants (irrespective of their views on family planning) and associated with encouraging good spousal communication. However, their appreciation of abstinence as a method seemed to be more in theory than practice, as many men also mentioned the difficulty in using this method to prevent pregnancies. Withdrawal, a method less frequently mentioned, was deemed “too difficult to apply.” Overall, men felt that male methods were too limited. Of the current 4 choices available to men, vasectomy was not considered a possibility; abstinence and withdrawal were too difficult to apply consistently and accurately; and male condoms had limited use, given lack of preference for this method in the community.

The fact that there are fewer contraceptive methods for men was mentioned by participants as part of the explanation for poor male engagement in family planning. Men thought that development of other male-controlled methods could encourage their involvement in family planning. Thus, if there was a disagreement between a husband and wife and the method was controlled by men, men would be in a better position to convince their disagreeing spouse. As one participant put it:

*… men can convince their wives to accept a method better than wives can convince their husbands.*

All men also had very strong opinions on current family planning services, including how the Togolese family planning campaign is run. The majority of participants believed that the campaign was not really for, nor did it target, men. They felt that although men are often the primary decision makers about family size and health services, family planning services and campaigns almost exclusively target women. They thought the family planning campaign had no specific information for men; awareness and education was mostly provided during antenatal care, which men do not attend; and the overall communication strategies employed were not directed at men. Some men cited existing work in their communities with community health workers but felt it was also largely directed at women.

## DISCUSSION

There is strong evidence to suggest that involving men in sexual and reproductive health programs can improve spousal communication, gender-equitable attitudes, and family planning use.^29^^–^^32^ Previous studies have also found that spousal communication is useful to foster contraceptive practice among couples.^33^^,^^34^ Given the significance of the man's role in deciding family size in sub-Saharan Africa, especially in francophone West Africa, there is a dearth of studies examining male engagement in family planning. Meanwhile, Ouagadougou Partnership member countries have aimed to reach 2.2 million additional modern contraceptive users by 2020, as well as revised action plans that incorporate scientific evidence from the region. Consequently, the findings from this study provide important insights into how men view family planning and how programs can further involve them in family planning efforts. Analyses highlighted 4 keys findings:

1. Socioeconomic motivations drive men's interest in family planning.
2. Men strongly disapprove of unilateral decision by women to use family planning.
3. Misconceptions surrounding modern methods can hinder support for family planning.
4. Limited method choice for men, insufficient venues to receive services, and few messages that target men all create barriers for male engagement in family planning.

Additionally, participants' views on family planning provide insights on how to tailor future family planning programs and campaigns to engage men. For example, the reasons cited by men who “believed in family planning” were consistent with the child's quantity-quality theory.^35^^,^^36^ This model assumes that parents' psychological satisfaction from their children is likely dependent on the investment parents make in the quality of children, in terms of their lifetime well-being, the quality of education received, and the quality of future job prospects. Therefore, belief in family planning is proportional to level of awareness of the high cost of raising quality children and the effect of the number of children on the quality of those children.

Men who had reservations about family planning comprised a segment of the population that is likely the most uninformed about modern methods, including side effects and implications for future fertility. From a programmatic perspective, focusing behavior change communication activities on this segment of the population would likely be extremely beneficial. The finding that all men wanted to be better informed about all methods is very encouraging. Interventions should educate men on the socioeconomic and health benefits of family planning while explaining the possible side effects and dispelling the myths around long-term negative health effects to the mother and child associated with use of modern contraceptive methods. More awareness on the value of contraceptive methods for both spacing and limiting would also be important.

The findings from the focus group discussions not only highlighted the family planning information that should be included in messaging and campaigns but also the best channels for delivering family planning information. Door-to-door dissemination of information was preferred, particularly with incorporation of “positive deviants,” a communication method that was also identified in another Togo study.^14^ Using positive deviance as an approach could be particularly beneficial to addressing misconceptions about infidelity and contraception that arose during the focus group discussions.

Men preferred door-to-door dissemination of information about family planning.

On the other hand, it is not clear from the analysis of the focus group discussions whether there is an opportunity to influence the men considered nonbelievers in family planning, as this group included men with fundamental differences of opinion specifically related to sex, sexuality, and sexual desire, and they refuted the need for modern methods to exercise control over sexual desire. Among some men's perspectives, sex is intended for procreation only. This group of men could potentially be reached by religious leaders, male motivators, or trained male peers, a strategy that has had some success in francophone West Africa.^9^^,^^16^^,^^37^

In addition to the importance of reaching men with information, the focus group findings highlighted the need to engage men as family planning clients, not just as the partners of women. A recent review of programming showed that men are not well served by current family planning programs, but when male-controlled methods were made available through interventions, uptake typically increased.^37^ The review also highlighted the need for more male-controlled methods. Focus group participants in this study found current methods, namely, male condoms and vasectomy, to be unsatisfactory, which is consistent with previous studies.^34^^,^^38^ Although using condoms is an effective means of birth control and protects against sexually transmitted infections, some couples claim that condoms create a barrier, which reduces pleasure during sexual intercourse.^38^ Likewise, although vasectomy is effective for birth control, its irreversibility constituted a principal barrier for men in this study.

Participants also linked the limited choices of effective and acceptable methods for men to the low level of male engagement in family planning. In addition, their opinion of the current national family planning campaign was negative and perceived as exclusive of men. From a gender norms perspective, this finding could be interpreted in two ways. Men might want to have more control over fertility regulation and having more male-controlled methods could be a way to achieve this control. Alternatively, they might simply feel excluded, given their perception that the family planning campaign is currently only targeting women in Togo, but also feel that if more male methods were available, the nature of the campaign would also focus on men. These findings were similar to the results from a rapid formative assessment of male engagement in family planning conducted in Togo as part of ongoing efforts around expanding no-scalpel vasectomy services.^14^ Themes from that assessment included the perceptions that family planning is only for women, there is a lack of methods available for men, services are not meeting men “where they are” (i.e., not at hours or locations convenient to men's schedules), there is a perceived lack of male providers offering family planning services for men, there is lack of communication about family planning among couples in the community, and social norms do not support use of family planning.^14^

It is important to note that program planners and policy makers must be sensitive to the former case where men's negative perception of family planning programs derives from a desire to be more in control of fertility. Engaging men in family planning requires addressing the norms that leave them thinking they are—and should be—in control. For example, as in the case of some of the responses during the focus group discussions, approval of leaving your wife or taking on another partner if she uses contraception is a sign of control. It is critical that male engagement in family planning should not come at the expense of women's agency for decision making.

Findings from this study, as well as other studies looking at male involvement in family planning, confirm the need for increased efforts to directly engage men in family planning discussions, campaigns, and services. It is clear that family planning interventions that target men provide an opportunity to improve progress in reaching goals surrounding contraception and fertility, particularly in countries where men retain decision-making power.

### Limitations

This study was not representative of Togo and focused on peri-urban and urban neighborhoods of Lomé, a capital city where family planning services are relatively available to women. There are also general limitations to qualitative research methods related to validity, reliability, and subjectivity.^39^ Translation of transcripts to English, a language common to the entire research team, may have resulted in details lost in translation; validity of the data may have been compromised in this process. Nonetheless, focus group discussions allowed us to better capture details and understand the nuances of engaging men in family planning in Togo, which might have been missed with more rigorous forms of quantitative data collection.

## CONCLUSION

Improving family planning programming to better involve men is imperative to increasing voluntary uptake of family planning among couples. This is particularly important in francophone West Africa, where men's influential role in family decision making is coupled with accelerated national goals of reaching new modern contraceptive users with services by 2020. Meeting these targets will not be possible without engaging men. Our theory of change for male engagement in family planning is based on the premise that involving men will lead to higher contraceptive use and better gender equality, as men are not only partners of contraceptive users but also family planning clients themselves and agents of change. However, many programs exclude men, thus failing to address their need for joint decision making in family planning choices.

Existing literature shows that there are many challenges to increasing male involvement in family planning.^20^ Attempts to address these challenges need to start by listening to men's concerns, misconceptions, and their views of their roles in family planning decision making, which this study provides insights on. To help build trust and facilitate open communication, family planning programs that encourage counseling of husbands and wives in their homes by community health workers, trusted men, or couples that have successfully used or are currently using family planning to achieve their desired family size will be important. Family planning campaigns should be tailored to also encompass men and be accompanied by increased availability of male methods to the extent possible. The integration of gender equity in family planning in Togo needs to be done, but in a careful manner. Finally, future studies should qualitatively explore the practices, characteristics, perceptions, and motivation that help men successfully engage in family planning, and then incorporate the findings in family planning programs and campaigns.

## Notes

**En français**

**Engagement des hommes dans la planification familiale : « Perspectives des hommes mariés dans la ville de Lomé, Togo »**

Les hommes participants à l’étude étaient en général favorable à l’utilisation de la contraception moderne dans le couple, en particulier pour des raisons socioéconomiques. Cependant, certains hommes ont manifesté des réserves, dues essentiellement à la perception que la planification familiale pourrait faciliter l’infidélité et la promiscuité. Ces mêmes hommes ont indiqué leur préférence pour que la décision en matière de planification familiale soit prise de commun accord entre les partenaires. Tous les hommes ayant participé à l’étude ont exprimé le désir d’apprendre davantage sur la planification familiale, avec une préférence pour les activités de sensibilisation menées par les agents de santé communautaires, d’autres hommes de confiance ainsi que par des utilisateurs actuels de la planification familiale.

**RÉSUMÉ **

Les programmes de planification familiale ont fait de grands progrès dans de nombreuses régions d'Afrique subsaharienne au cours de la dernière décennie, même si l'Afrique de l'Ouest francophone reste encore « à la traîne ». L'accent mis sur l'engagement des hommes pourrait aboutir à de meilleurs résultats, en particulier dans les pays structurés autour de sociétés patriarcales fortes. Peu d'études en Afrique de l'Ouest francophone ont examiné les attitudes des hommes en matière de planification familiale du point de vue des hommes eux-mêmes. Pourtant ces preuves sont nécessaires pour le développement de projets de planification familiale incluant des activités ciblant les hommes. Cette étude qualitative, menée en 2016 par USAID/WA-Evidence for Development, a exploré les attitudes de 72 hommes mariés âgés de 18 à 54 ans à travers 6 groupes de discussions dans la capitale du Togo, Lomé. Les participants à l’étude incluaient des travailleurs professionnels ainsi que d’autres travailleurs « qualifiés » et « non qualifiés ». Les résultats indiquent que les hommes ont des points de vue spécifiques sur la planification familiale ; lesquels points de vue sont basés sur leurs connaissances et de leur compréhension du « comment et du pourquoi » les femmes pourraient utiliser une méthode moderne de contraception. Alors que certains hommes ont indiqué avoir des réserves, fondées ou non, il y avait une réaction extrêmement favorable pour discuter de la planification familiale et même de participer à la prise de décisions et la mise en œuvre des services connexes. Les quatre principales conclusions des analyses des groupes de discussions ont été les suivantes : (1) les motivations socioéconomiques poussent les hommes à s'intéresser à la planification familiale ; (2) les hommes désapprouvent fortement les décisions unilatérales de leurs femmes d'adopter une méthode de planification familiale ; (3) les idées fausses entourant les méthodes modernes peuvent entraver les efforts de soutien à la planification familiale ; et (4) un choix limité de méthodes pour les hommes, l’insuffisance de sources et lieux pour recevoir des services, et peu de messages spécifiques ciblant les hommes sont autant de barrières pour leur engagement dans la planification familiale. Les futures initiatives engageant les hommes dans les programmes de planification familiale devraient accorder une attention particulière à leurs préoccupations spécifiques, aux « idées fausses » et à leurs rôles dans la prise de décision familiale. Les interventions devraient permettre aux hommes de mieux appréhender les avantages socio-économiques et sanitaires de la planification familiale tout en expliquant les effets secondaires possibles et en dissipant les mythes. Pour aider à établir la confiance et faciliter la communication ouverte dans la famille, les programmes de planification devraient encourager le counseling des maris et leurs épouses dans leur foyer par les agents de santé communautaires, les hommes de confiance ou les couples qui utilisent ou ont utilisé avec succès les services de planification familiale pour atteindre leurs objectifs en matière de famille.

## Notes

**Peer Reviewed**

**Funding:** This study was conducted with data collected through the Evidence for Development Project funded by the United States Agency for International Development West Africa. The points of view in this study are solely those of the authors and do not necessarily reflect the views of United States Agency for International Development.

**First Published Online:** May 9, 2018
